# Simulated Investigation in Wormhole Expansion Law of Gelling Acid Etching and Its Influencing Factors in Deep Carbonate Reservoirs

**DOI:** 10.3390/gels8080470

**Published:** 2022-07-27

**Authors:** Mingwei Wang, Wen Zhou, Song Li, Wen Wu

**Affiliations:** 1 State Key Laboratory of Oil and Gas Reservoir Geology and Exploitation, Chengdu University of Technology, Chengdu 610059, China; 201999010137@swpu.edu.cn (M.W.); zhouwen62@cdut.edu.cn (W.Z.); 2 School of Oil & Natural Gas Engineering, Southwest Petroleum University, Chengdu 610500, China; 3 Engineering Research Institute of PetroChina Southwest Oil, Gasfield Company, Chengdu 610017, China; 4 Southwest Oil and Gas Field Company of CNPC, Chengdu 610041, China; wuwen2016@petrochina.com.cn

**Keywords:** reservoir stimulation, gelled acidizing, wormhole propagation, carbonate rocks, simulation

## Abstract

Acidizing with gelling acid is the key technology in developing a carbonate reservoir successfully. It is difficult for the laboratory to carry out the radial displacement experiment with a large rock core. It is necessary to establish the gelling acid wormhole expansion model under the radial conditions, simulate the gelling acid wormhole expansion law under the radial conditions, optimize the construction parameters, and provide the basis for the optimal design of carbonate reservoir matrix acidizing. The research objective is to simulate the gelling acid etching wormhole expansion in a deep carbonate reservoir and make clear its influencing factors, which are helpful for reservoir stimulation. The mathematical model of gelling acid wormhole expansion was established, considering the influence of pore microscopic characteristics on acid flow and acid rock reaction. The simulation results indicated that viscosity, surface reaction rate, and hydrogen ion diffusion coefficient have different effects on gelling acid etching wormhole. The spatial distribution of pores determines the trend of gelling acid solution and thus the shape of the armhole. Perforation completion has a significant impact on the expansion of gelling acid etching wormhole. The wormhole extends forward along the perforation hole, and perforation increases the length of the wormhole. This wormhole expansion law is very suitable in situations where a deep carbonate reservoir is needed for gelling acid fracturing.

## 1. Introduction

Gelling acidizing is carried out under the radial conditions of the wellbore. It is difficult for the laboratory to carry out the radial displacement experiment with a large rock core. It is necessary to establish the gelling acid wormhole expansion model under the radial conditions, simulate the gelling acid wormhole expansion law under the radial conditions, optimize the construction parameters, and provide the basis for the optimal design of matrix gelling acidizing [1]. 

At present, there are a capillary model, network model, single pore model, two-scale model, and lattice Boltzmann model [2,3,4,5], they also investigate the dynamics of model polymer networks formed by the condensation of linear poly precursor and PDCA ligand and the subsequent complexation with different metal ions at various pH values and oxidation states [6]. Huang et al. (2000) [7] proposed a single wormhole model to represent the wormhole via a cylindrical tube and considered the effects of fluid leakage and reaction kinetics on wormhole growth. Panga et al. (2004, 2005) [8,9] established the two-scale continuous model to explore the wormhole’s initiation and propagation in acidizing carbonate reservoirs. The model considered the effect of reaction rate regime, wormhole density, and dimension on wormhole expansion. Cohen et al. (2007) [10] extended this model to radial flow to study the effect of flow geometry on the PVBT curve. They showed that the optimum injection rate in radial flow is higher than that of linear flow. Akanni and Nasr-El-Din (2015, 2016) [11,12,13] confirmed that gelling acidizing can reduce the reservoir skin factor by injecting gelling acid, which can consequently be created in the wormholes. The wormhole models were developed to simulate the wormhole patterns, which were observed in the experiment and account for physical phenomena of acidizing such as diffusion, convection, pore dissolution, and rock heterogeneities very well. They were also extended to radial coordinate or spatially correlated pore distributions (Liu et al., 2012; Zhang et al., 2014; Mou et al., 2015) [14,15,16]. Qiu et al. (2018) [17] completed a series of radial acid injection and linear acid flow experiments, which are conducted at various injection rates while maintaining the same operating conditions. It is found that the pore volume to breakthrough values of the linear acid flow case is lower for the radial acidizing case. QI Dan et al. (2019) [18] established a calculation model of wormhole to analyze wormholes quantitatively, optimize assumed gelling acid volume, and pumping rate and obtain the best production rate, which can be used to describe the method of the fractal. sensitivity analysis of influencing parameters and field acidification calculation was carried out. L Wang et al. (2019) [19] conducted research on wormhole propagation under radial flowing conditions in naturally fractured carbonates, natural fracture models were established by using the Monte Carlo method, and extensive numerical simulation was conducted to study wormhole characteristics considering the effects of natural fractures. N.Z. Liu et al. (2016) [20] developed a novel model to simulate wormhole propagation through VES acidizing. The simulation results showed that the injection velocity of VES acid was a significant factor for wormhole propagation. They also considered that the diversion effectiveness of wormhole was determined by the effect of both viscosifying and wormhole. W Wei et al. (2020) [21] established a two-scale continuum model in a 3D compositional reservoir simulator considering fractures by EDFM (Embedded Discrete Fracture Model), which describes convection and dispersion while IPhreeqc is responsible for the dissolution calculation both in the matrix and on the fracture surface.

N Qi et al. (2019) [22] studied the wormholes of the two-scale continuum model and pseudo-fracture model considering different fractures, and it is found that the gelling acid can be concentrated and accelerated penetrating the formation if fractures parallel to the injection direction. The effect of straight fractures and arc fractures on wormholes was defined. Mustafa et al. (2022) [23] established acid efficiency curves which included different rock lithologies (chalk, limestone, and dolomite) that react with gelling acid at different injection rates. The rock surface hardness at ambient pressure was measured by the impulse hammer technique, and the dynamic Young’s modulus and Poisson ratio were measured through acoustic measurements at high confining pressures. Ghommem et al. (2015) [24] developed and validated a predictive model for carbonate acidizing, benchmarked by linear core flooding experiments, which used the two-scale continuum approach to simulate gelling acid flowing and wormhole propagation in carbonate rocks. H Yoo et al. (2021) [25] developed the wormhole propagation behavior to optimize an efficient acid treatment in carbonate acidizing, which used micro X-ray computerized tomography (CT) to observe wormhole visualization clearly. It is found that the wormhole diameters were gradually reduced along with the wormhole propagation axis in all cases, and the wormhole diameter tended to increase according to the higher acid concentration and permeability. A continuum two-scale model for linear and radial flow geometry was developed to simulate dissolution patterns. The simulation results showed that the in-situ generated hydrochloric at low injection rates could create longer wormholes with less pore volume to breakthrough, due to low face dissolution [26]. 

Research on gelling acid for reservoir stimulation has focused on middle and deep carbonate reservoirs and other areas [27,28,29,30]. Yuman Wu et al. (2022) [31] proposed a betaine-based gel used for hydraulic fracturing, it was proved that KCl improves the temperature resistance and increases the viscosity of the optimized fracturing fluid, and the viscoelastic surfactant gel had high shear resistance and high sand-carrying performance. Fei Ding et al. (2022) [32] developed a PHRO gel that was composed of gelatinizing agents, crosslinking agents, and crosslinking promoting agents (oxalic acid). The performance evaluation showed that it has good salt-resistance properties and is suitable for conformance control of low-temperature and high-salinity reservoirs.

At present, more two-scale models are used. This model can take into account the physical phenomena such as gelling acid flow, gelling acid rock reaction, and pore structure change, and can better simulate the gelling acid wormhole morphology obtained in the laboratory. Double scale refers to the Darcy scale and pore scale, that is, the flow is calculated on the Darcy scale; gelling acid rock reaction considers the characteristics of the pore scale, and the reaction speed of gelling acid rock is controlled by specific surface and mass transfer speed, which is the affected by pore size. Therefore, it is necessary to establish a model on a pore scale and consider the influence of micro characteristics on flow and reaction.

## 2. Results and Discussion

### 2.1. Effect of Injection Rate on the Morphology of Vermicular Foramen

**Figure 1:**
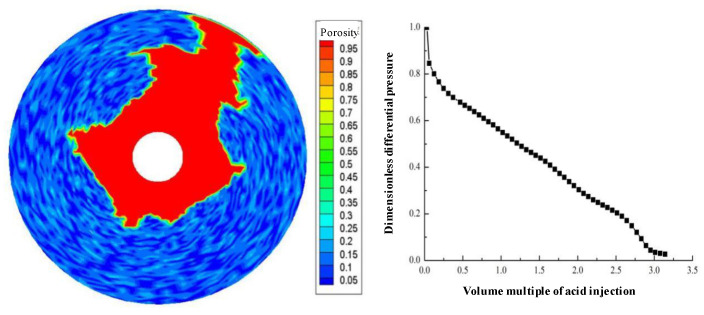
Change of dissolution form and differential pressure with gelling acid injection volume (gelling acid injection speed 0.0065 cm/min).

**Figure 2:**
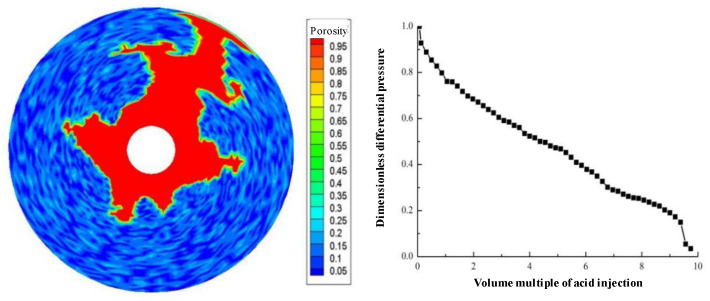
Change of dissolution form and differential pressure with gelling acid injection volume (gelling acid injection speed 0.0135 cm/min).

**Figure 3:**
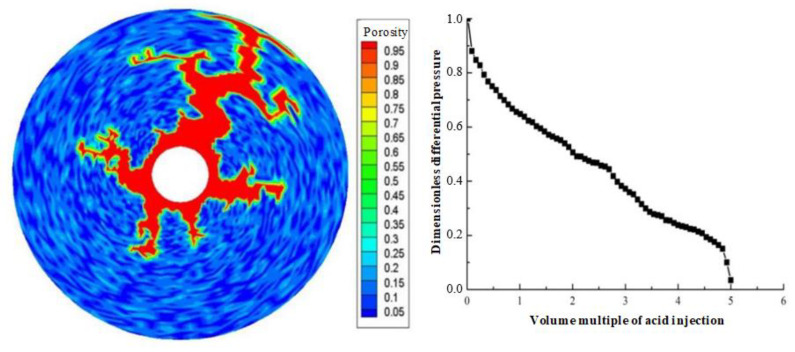
Change of dissolution form and differential pressure with gelling acid injection (gelling acid injection speed 0.035 cm/min).

**Figure 4:**
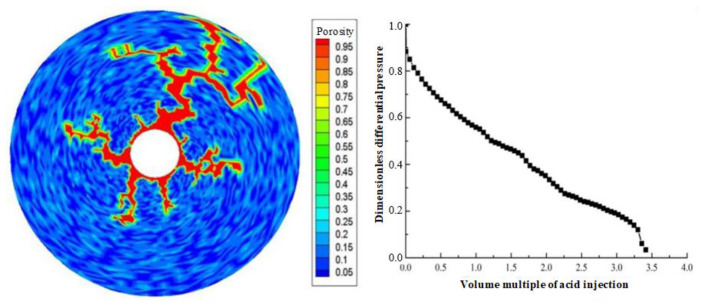
Change of dissolution form and differential pressure with gelling acid injection (gelling acid injection speed 0.065 cm/min).

**Figure 5:**
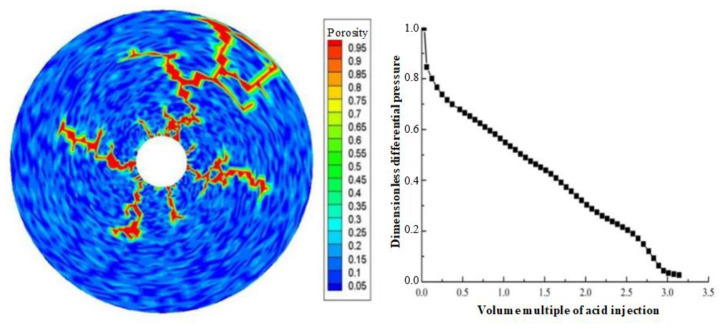
Change of dissolution form and differential pressure with gelling acid injection (gelling acid injection speed 0.65 cm/min).

**Figure 6:**
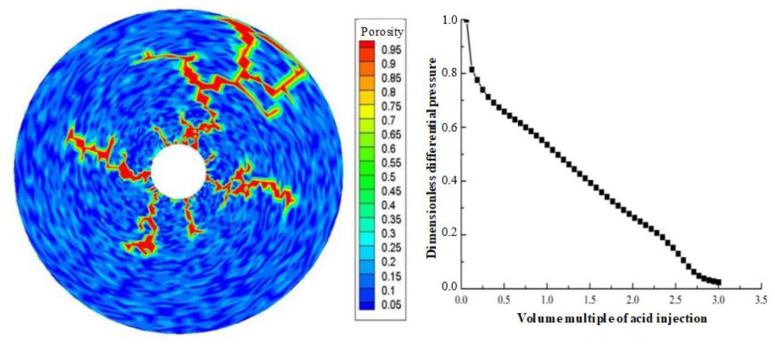
Change of dissolution form and differential pressure with gelling acid injection (gelling acid injection speed 1.35 cm/min).

**Figure 7:**
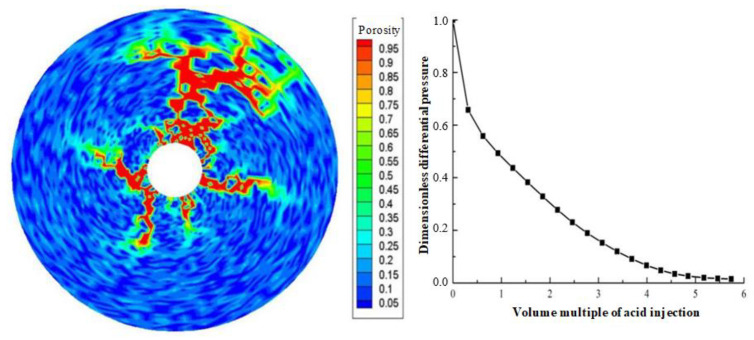
Change of dissolution form and differential pressure with gelling acid injection (gelling acid injection speed 6.5 cm/min).

**Figure 8:**
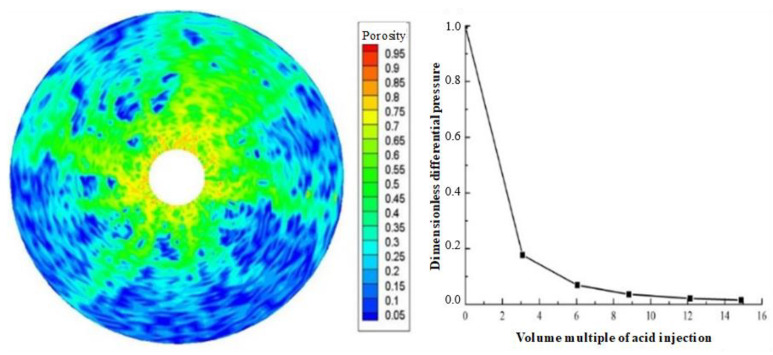
Change of dissolution form and differential pressure with gelling acid injection (gelling acid injection speed 65 cm/min).

Displacement (injection rate) is an important parameter in gelling acidizing construction. It is necessary to study the impact of injection rate on wormhole expansion. Figure 1, Figure 2, Figure 3, Figure 4, Figure 5, Figure 6, Figure 7 and Figure 8 show the changes in wormhole morphology and displacement differential pressure under different gelling acid injection rates. Dimensionless differential pressure is defined as the ratio between the differential pressure at both ends of the core and the initial value during gelling acid injection, which reflects the flow of gelling acid in the rock core. Zero dimensionless differential pressure means that the gelling acid breaks through the core.

The gelling acid injection rate increased from 0.0065 cm/min to 65 cm/min, and the model simulation results are shown in Figure 1, Figure 2, Figure 3, Figure 4, Figure 5, Figure 6, Figure 7 and Figure 8. The gelling acid corrosion morphology showed the phenomena observed in the experiment: surface corrosion, main wormhole, branch wormhole, and uniform corrosion. When the gelling acid injection speed is very small because the diffusion effect of hydrogen ions in the gelling acid solution is greater than the convection effect, the hydrogen ions are more likely to diffuse upward (i.e., the hole wall), which prevents the gelling acid solution from dissolving the wormhole, and there is less gelling acid reaching the front end of the wormhole. Under this injection speed, increasing the wormhole size is not conducive to increasing the wormhole length, and the excessive dissolution of rocks on the well wall may easily cause the collapse of the well wall. With the increase in gelling acid injection speed, as are shown in Figure 1, Figure 2, Figure 3, Figure 4, Figure 5, Figure 6, Figure 7 and Figure 8, the convection effect of hydrogen ions gradually increases, but it is still less than the diffusion effect. More gelling acid is consumed on the wall of the earthworm hole. However, due to the enhancement of convection, the dissolution form gradually transits to the earthworm hole. As shown in Figure 5, when the gelling acid injection speed is relatively moderate, the diffusion and convection of hydrogen ions are equivalent. More gelling acid reaches the front end of the wormhole, and the gelling acid reaching the front end of the wormhole not only completely reacts with the rock to promote the growth of the wormhole, but also forms the main wormhole. With the further increase in gelling acid injection speed, the convection effect of hydrogen ions is gradually greater than the diffusion effect. The gelling acid liquid flows forward before it has time to completely react with the encountered rock. The wormholes formed due to the influence of heterogeneity are branched. When the gelling acid injection speed is high (Figure 8), the convection effect of hydrogen ions is much greater than the diffusion effect. The gelling acid liquid flows into the pores before consumption, so that live gelling acid can be obtained everywhere, forming a more uniform dissolution form, and the efficiency of removing pollution is low.

**Figure 9:**
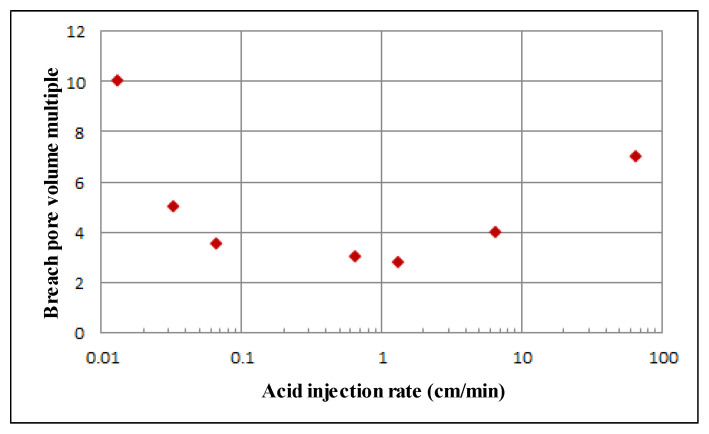
Effect of injection rate on breakthrough volume multiple.

The effect of injection rate on breakthrough volume multiple is shown in Figure 9. The trend of this curve is similar to that obtained in the laboratory. When the injection speed gradually increases from the lowest, the breakthrough pore volume multiple first decreases and then reaches a minimum value. The injection speed corresponding to this point is called the optimal injection speed and then continues to increase the injection speed, resulting in the slow increase in breakthrough pore volume multiple. On the left side of the optimal injection speed, the breakthrough pore volume multiple increases sharply with the decrease in the injection speed, while on the right side of the optimal injection speed, the breakthrough pore volume increases slowly with the increase in the injection speed. In actual construction, it is difficult to obtain the optimal injection speed. For the sake of safety, try to keep the injection speed on the right side of the optimal injection speed. If it is on the left, the injection speed fluctuates, which has a great impact on the breakthrough pore volume. On the right side of the optimal injection velocity, the fluctuation of injection velocity has little effect on the breakthrough pore volume. The simulation results show that for this simulation condition, the optimal injection rate is about 1 cm/min, and the corresponding breakthrough pore volume multiple is about 1.4. The simulation of gelling acid wormhole expansion under radial conditions shows that it is necessary to select an appropriate injection rate. The injection rate is too low, the gelling acid solution dissolves near the wellbore, the action distance is very short, and the plugging removal effect cannot be achieved. The gelling acid injection speed is too fast, forming uniform corrosion, unable to form wormhole, and the transformation effect is not good. At the optimal injection rate, the main wormhole is formed, and the seepage resistance in the wormhole can be ignored. The wormhole passes through the pollution zone to remove the pollution, which is equivalent to expanding the wellbore radius and increasing the production capacity.

### 2.2. Influence of Gelling Acid Properties

**Figure 10:**
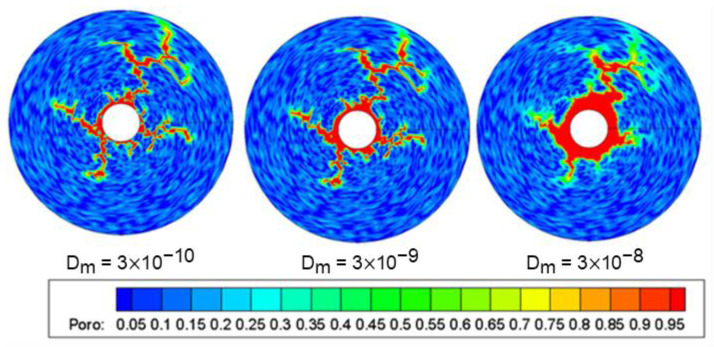
Effect of hydrogen ion diffusion coefficient on wormhole morphology.

The properties of gelling acid solution mainly include these aspects: viscosity, surface reaction rate, and hydrogen ion diffusion coefficient. In matrix gelling acidification, to facilitate injection, low viscosity gelling acid solution is used, and viscosity is not the main factor. The reaction between carbonate rock and gelling acid is mainly affected by the mass transfer rate, and the hydrogen ion diffusion coefficient is the controlling factor of the mass transfer coefficient. Therefore, only the influence of hydrogen ion diffusion coefficient is analyzed. Figure 10 shows the effect of the hydrogen ion diffusion coefficient on wormhole morphology, and other conditions are the same. When the diffusion coefficient is low, the wormhole is fine. With the increase in the diffusion coefficient, the wormhole near the well becomes thicker, and more gelling acid is consumed near the well; when the diffusion coefficient is high, there is more dissolution in the wellbore wall and the wormhole is thicker. When the diffusion coefficient is high, to form the main wormhole, the injection speed needs to be increased to match the injection speed with the reaction.

### 2.3. Effect of Pore Space Distribution on Wormhole Morphology

**Figure 11:**
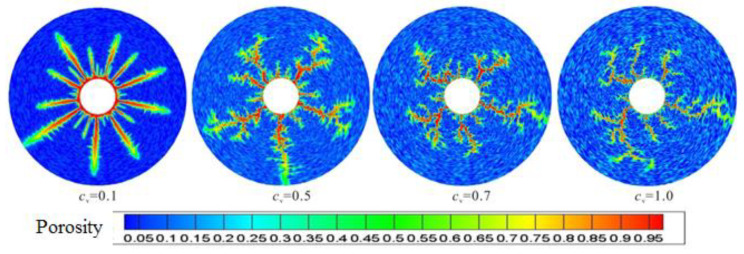
Effect of pore space distribution on wormhole morphology.

The spatial distribution of pores determines the trend of gelling acid solution and thus the shape of the wormhole. *C_v_* is the coefficient of variation of porosity. The smaller the *C_v_* is, the weaker the heterogeneity is, on the contrary, it is about strong. When *C_v_* is 1, more high porosity places can be seen on the pore distribution map. Figure 11 shows the wormhole morphology when the heterogeneity gradually becomes stronger from left to right. For the mean value case, there are more wormholes, and each wormhole is similar; when the heterogeneity is strong, the non-uniform competition of gelling acid flow and reaction is stronger, and the numerical simulation of the main wormhole is less. When *C_v_* = 0.7 and 1, the simulated wormhole is more like the wormhole observed in the experiment.

**Figure 12:**
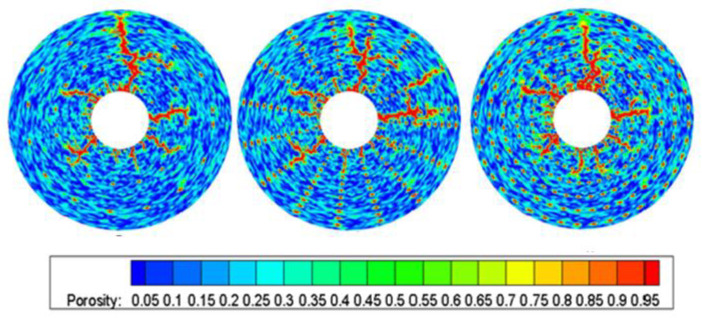
Effect of hole distribution on wormhole expansion.

Figure 12 shows the wormhole shape when adding holes. When the holes are dispersed, the influence of the convection field is small, so it has little influence on the direction of wormhole. If the holes are continuously distributed, the gelling acid easily flows along hole distribution area, thus affecting the wormhole shape.

**Figure 13:**
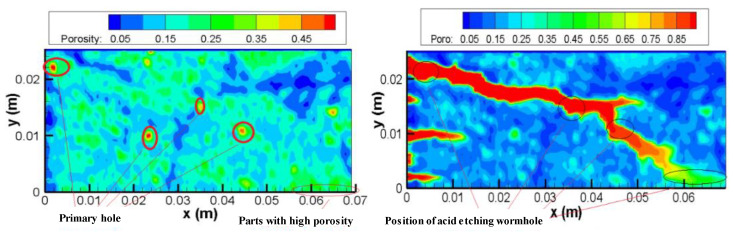
Effect of pore space distribution on the expansion of gelling acid wormhole.

Figure 13 left shows the real pore spatial distribution of the core. The figure shows that the core porosity distribution is not random, but spatial correlation. There is a high seepage band from the core inlet to the outlet. The figure on the right shows the simulated gelling acid etching wormhole diagram. In the simulation, the wormhole is formed from the upper left and gradually expands to the lower right corner along the high seepage zone. The wormhole path is the distribution area of the high seepage zone, indicating that the pore space distribution determines the direction of the wormhole.

### 2.4. Effect of Perforation on Wormhole Shape

**Figure 14:**
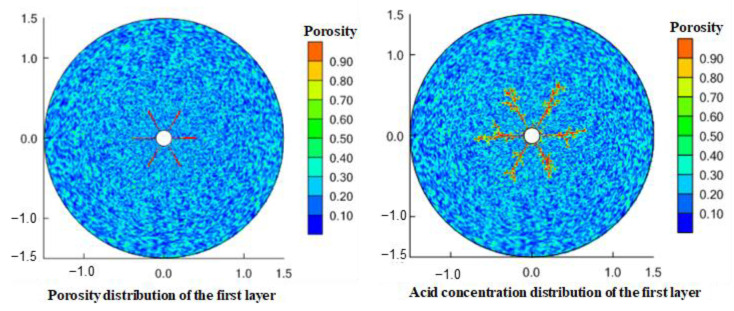
Wormhole shape during 60° phase angle perforation.

**Figure 15:**
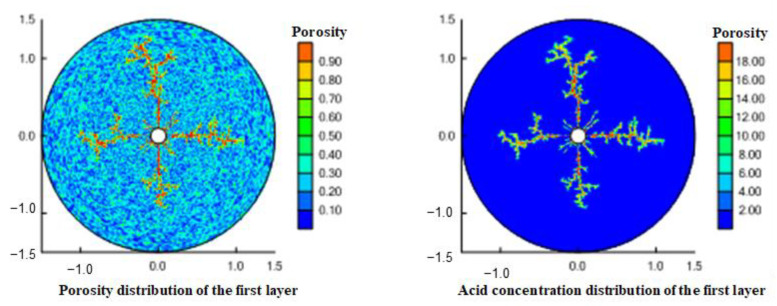
Wormhole shape during 90° phase angle perforation.

The perforation hole is a macro channel. For the perforation completion wellbore, the seepage resistance in the hole can be ignored. The injected gelling acid flows to the formation through the perforation hole and bypasses the perforation coverage area. Therefore, the perforation completion will have a significant impact on the expansion of the gelling acid wormhole. Figure 14 and Figure 15 simulate the shape of gelling acid-etched wormholes under perforation conditions. Wormholes are formed and expanded at the front end of perforations. When perforating with a 60° phase angle, 6 wormholes expand outward, and when perforating with a 90° phase angle, 4 wormholes expand outward. As the outward radius increases, the overflow cross-sectional area increases, the gelling acid in the wormhole is filtered around, and the wormhole branches. The more outward, the more branches; the larger the perforation phase angle is, the less the initial expanded wormhole digital modulus is, and the wormhole branch increases because the gelling acid fluid loses more to the surrounding. Through comparative analysis, it is found that the 6 wormholes with a 60° phase angle perforation expand outward shorter than the 4 wormholes with a 90° phase angle perforation. The perforation hole is equivalent to a very large wormhole. The perforation completion is when the wormhole extends forward along the perforation hole, and the perforation increases the length of the wormhole.

## 3. Conclusions

(1) The mathematical model of gelling acid etching wormhole is established, and the expansion experiment of wormhole in the radial core is carried out with a real core to verify the correctness of the model.

(2) The gelling acid injection rate increased from 0.0065 to 65 cm/min, corresponding to the gelling acid corrosion morphology of wormhole: surface corrosion, main wormhole, branch wormhole, and uniform corrosion.

(3) Viscosity, surface reaction rate, and hydrogen ion diffusion coefficient have different effects on gelling acid etching wormhole. Viscosity and surface reaction rate are not the main factors. The reaction between carbonate rock and gelling acid is mainly affected by mass transfer rate. The hydrogen ion diffusion coefficient determines the mass transfer coefficient. When the diffusion coefficient is low, the wormhole is fine; with the increase in the diffusion coefficient, the wormhole near the well becomes thicker, and more gelling acid is consumed near the well. When the diffusion coefficient is high, there is more dissolution in the wellbore wall and the wormhole is thicker. When the diffusion coefficient is high, to form the main wormhole, the injection speed needs to be increased to match the injection speed with the reaction.

(4) The spatial distribution of pores determines the trend of gelling acid solution and thus the shape of the armhole. The smaller the coefficient of variation of porosity is, the weaker the heterogeneity is, and the more the numerical models of wormholes are, and the wormholes are similar. When the heterogeneity is strong, the non-uniform competition of gelling acid flow and reaction is stronger, and the numerical simulation of the main wormhole is less.

(5) Perforation completion has a significant impact on the expansion of gelling acid etching wormhole. The wormhole extends forward along the perforation hole, and perforation increases the length of the wormhole. When 60° phase angle perforation, 6 wormholes expand outward, and when 90° phase angle perforation, 4 wormholes expand outward. As the outward radius increases, the overflow cross-sectional area increases, the gelling acid in the wormhole is filtered around, and the wormhole branches. The more outward, the more branches. The larger the perforation phase angle is the less the initial expanded wormhole digital modulus is, and the wormhole branch increases.

## 4. Materials and Methods

Two-scale continuum model was considered to simulate acid flow, acid-rock reaction, porosity change, and wormhole expansion during acidizing of carbonate reservoirs. The detail of the model has been discussed by Panga et al. (2005). The model of Gelling Acid Etching Wormhole that we established is considering the influence of pore microscopic characteristics on acid flow and acid rock reaction. It is considered a natural transition between porous media flow and Stokes free flow in wormholes; fractures are influenced by pressure and velocity fields.

### 4.1. Continuity Equation

**Figure 16:**
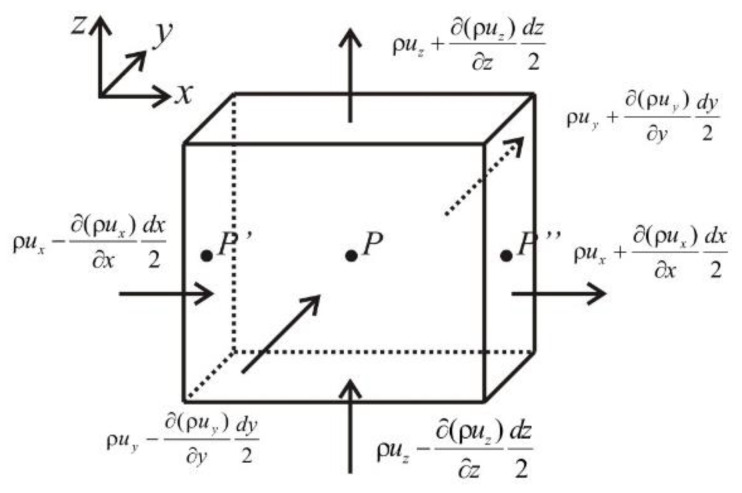
Schematic diagram of micro hexahedron unit.

According to the fluid mass balance, inflow term-outflow term + source-sink term = cumulative term. As shown in Figure 16, the micro hexahedron unit is taken in the stratum, and the components of mass velocity of point *P* in unit on the three coordinates are *ρu*_x_, *ρu*_y_ and *ρu*_z_, then the mass flow of point *P′* in the x-direction is:

$$\rho u_{x}-\frac{\partial (\rho u_{x})}{\partial x}\frac{dx}{2}$$

The mass flow flowing in through the face of *P′* in *dt* time is:

$$[\rho u_{x}-\frac{\partial (\rho u_{x})}{\partial x}\frac{dx}{2}]dydzdt$$

Similarly, the mass flow of the plane where *P*″ is located in the x-direction is

$$[\rho u_{x}+\frac{\partial (\rho u_{x})}{\partial x}\frac{dx}{2}]dydzdt$$

Then the mass flow difference of hexahedron flowing in and out from x-direction in *dt* time is

$$-\frac{\partial (\rho u_{x})}{\partial x}dxdydzdt$$

Similarly, the mass flow difference of inflow and outflow from *y* and *z* directions in *dt* time is

$$-\frac{\partial (\rho u_{y})}{\partial y}dxdydzdt\;-\frac{\partial (\rho u_{z})}{\partial z}dxdydzdt$$

Then the total mass flow difference of inflow and outflow in the hexahedron in *dt* time is

$$-[\frac{\partial (\rho u_{x})}{\partial x}+\frac{\partial (\rho u_{y})}{\partial y}+\frac{\partial (\rho u_{z})}{\partial z}]dxdydzdt$$

The total change of fluid mass in *dt* time is

$$\frac{\partial (\rho \varphi )}{\partial t}dxdydzdt$$

where, *ρ* is the fluid density, kg/m^3^; *ϕ* is formation porosity, %.

Then the total mass change of hexahedron in *dt* time equals to the mass difference between inflow and outflow of hexahedron in *dt* time:

(1) $$\frac{\partial u_{x}}{\partial x}+\frac{\partial u_{y}}{\partial y}+\frac{\partial u_{z}}{\partial z}+\frac{\partial \varphi }{\partial t}=0$$

The above formula is the continuity equation of incompressible fluid seepage in porous media. If the *z*-direction is ignored and the above formula is converted to the polar coordinate system, the two-dimensional continuity equation can be obtained as:

(2) $$\frac{1}{r}\frac{\partial (ru_{r})}{\partial r}+\frac{1}{r}\frac{\partial u_{\theta }}{\partial \theta }+\frac{\partial \varphi }{\partial t}=0$$

where, *u_r_* and *u_θR_* are speed in direction *r* and *θ* direction, m/s.

### 4.2. Equation of Motion

The motion equation is the Darcy seepage equation, and the motion equation in the polar coordinate system is as follows:

(3) $$\left(u_{r},u_{\theta })=-\frac{K}{\mu_{a}}(\frac{\partial p}{\partial r},\frac{1}{r}\frac{\partial p}{\partial \theta }\right)$$

where, *K* is the rock permeability, μm^2^; *μ*_a_ is gelling acid viscosity, mPa·s; *p* is reservoir pressure, MPa.

### 4.3. Gelling Acid Distribution Equation

The flow of gelling acid in porous media is affected by both convection and diffusion. Similar to the derivation of the continuity equation, suppose a hexahedral element, the mass flow caused by diffusion at point *P* is *u*_i_, and then the x-direction passes through *P′*

$$u_{i}-\frac{\partial u_{i}}{\partial x}\frac{dx}{2}$$

The mass flow of the outflow unit through the face of *P″* is:

$$u_{i}+\frac{\partial u_{i}}{\partial x}\frac{dx}{2}$$

Then the mass flow difference of inflow and outflow units in the *x* direction is:

$$-\frac{\partial u_{i}}{\partial x}dx$$

Similarly, the mass flow difference of inflow and outflow units in *y* and *z* directions is:

$$-\frac{\partial u_{j}}{\partial y}dy,-\frac{\partial u_{k}}{\partial z}dz$$

The mass flow of point *P* caused by convection in the x-direction is *u*_x_*C*_f_, and the mass flow difference in the X direction through the *P′* and *P″* planes is:

$$-\frac{\partial (u_{x}C_{f})}{\partial x}dx$$

where, *C*_f_ is gelling acid concentration, mol/L.

Similarly, the mass flow difference in the *y*, *z* direction of inflow and outflow unit is:

$$-\frac{\partial (u_{y}C_{f})}{\partial y}dy,-\frac{\partial (u_{z}C_{f})}{\partial z}dz$$

It can be seen from the above that the mass change caused by convection and diffusion in *dt* time is:

$$-(\frac{\partial u_{i}}{\partial x}+\frac{\partial u_{j}}{\partial y}+\frac{\partial u_{k}}{\partial z})dxdydzdt-[\frac{\partial (u_{x}C_{f})}{\partial x}+\frac{\partial (u_{y}C_{f})}{\partial y}+\frac{\partial (u_{z}C_{f})}{\partial z}]dxdydzdt$$

The mass change caused by convection-diffusion in *dt* time must be equal to the total mass change in *dt* time:

$$-(\frac{\partial u_{i}}{\partial x}+\frac{\partial u_{j}}{\partial y}+\frac{\partial u_{k}}{\partial z})-[\frac{\partial (u_{x}C_{f})}{\partial x}+\frac{\partial (u_{y}C_{f})}{\partial y}+\frac{\partial (u_{z}C_{f})}{\partial z}]=\frac{\partial (\varphi C_{f})}{\partial t}$$

According to Fick’s law and considering the effective diffusion in porous media, the above formula can be written as:

(4) $$\nabla (\varphi D_{e}\cdot \nabla C_{f})-\nabla (UC_{f})=\frac{\partial (\varphi C_{f})}{\partial t}$$

where, *D*_e_ is the effective diffusion coefficient of the gelling acid solution, m^2^/s.

Due to the gelling acid rock reaction, the quality change caused by the chemical reaction must be considered. Here, assuming that the gelling acid flow to the wall of porous media completely reacts with the rock, the gelling acid concentration on the wall of the pore can be regarded as 0, and the gelling acid concentration in the pore is the original gelling acid concentration. In this way, there is a concentration gradient from the center of the pore to the wall of the pore. The size of the concentration gradient depends on the mass transfer rate from the fluid mass transfer to the fluid-solid surface and the reaction rate on the pore surface. If the reaction speed is less than the mass transfer speed, the concentration gradient can be ignored. At this time, the speed of the whole gelling acid rock reaction is controlled by the reaction speed of the liquid-solid interface. When the reaction rate is greater than the mass transfer rate, a large concentration gradient appears in the pores, which can be described by a simple concentration difference, as follows:

(5) $$k_{c}\left(C_{f}-C_{s}\right)=k_{s}C_{s}=R(C_{s})$$

where, *k*_c_ is the mass transfer coefficient of the gelling acid solution, m/s; *C*_s_ is gelling acid concentration on liquid-solid surface, mol/L; *k*_s_ is the gelling acid rock reaction rate constant, m/s.

Therefore, the mass change caused by a reaction can be expressed as:

$$k_{c}a_{v}\left(C_{f}-C_{s}\right)$$

where, *a*_v_ is specific surface area, m^−1^.

Bring the above formula into Equation (4):

(6) $$\nabla (\varphi D_{e}\cdot \nabla C_{f})-\nabla (UC_{f})-k_{c}a_{v}\left(C_{f}-C_{s}\right)=\frac{\partial (\varphi C_{f})}{\partial t}$$

The above formula is the gelling acid mass balance equation considering convection, diffusion, and reaction, which is converted to the polar coordinate system:

(7) $$\begin{array}{l} \frac{\partial (\varphi C_{f})}{\partial t}+\frac{1}{r}\frac{\partial }{\partial r}(ru_{r}C_{f})+\frac{1}{r}\frac{\partial }{\partial \theta }(u_{\theta }C_{f})\\ =\frac{1}{r}\frac{\partial }{\partial r}(r\varphi D_{er}\frac{\partial C_{f}}{\partial r})+\frac{1}{r}\frac{\partial }{\partial \theta }(\frac{\varphi D_{e\theta }}{r}\frac{\partial C_{f}}{\partial \theta })-k_{c}a_{v}(C_{f}-C_{s}) \end{array}$$

where, *D*_er_ and *D*_eθ_ are, respectively, effective diffusion coefficients in the *r* and *θ* direction, m^2^/s.

Equation (5) can be converted into the following formula:

(8) $$C_{s}=\frac{C_{f}}{1+k_{s}/k_{c}}$$

*k*_s_ << *k*_c_, when the reaction rate constant is much less than the mass transfer coefficient, *C*_s_ approximately equal to *C*_f_; *k*_s_ >> *k*_c_, when the reaction rate constant is much greater than the mass transfer coefficient, *C*_s_ approximately equal to 0. Since the reaction rate constant is approximately determined for a specific gelling acid solution, the control conditions of the whole gelling acid rock reaction are determined by the mass transfer coefficient. For porous media, due to the existence of heterogeneity and gelling acid rock reaction, the mass transfer coefficient also changes with time and space.

### 4.4. Model Validation 

**Figure 17:**
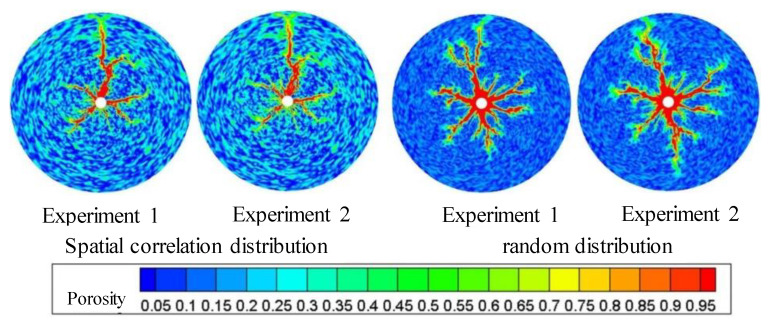
Simulated etching wormhole morphology obtained by CT scanning the rock end face gelled acid treated.

**Figure 18:**
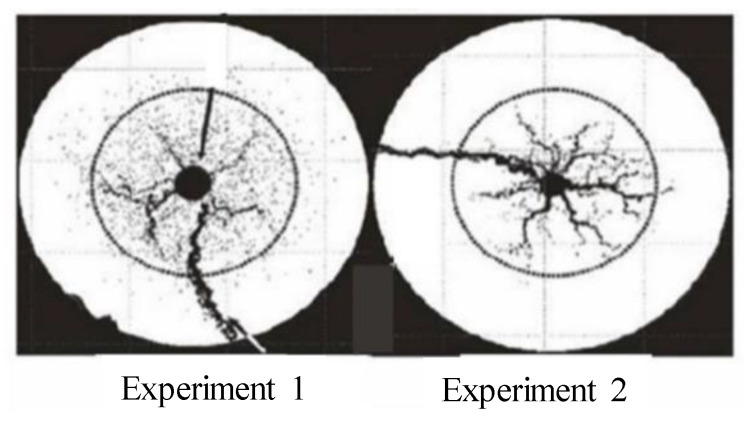
Radial wormhole morphology (adapted with permission from Ref. [27]. 2007, copyright Tardy et al.).

To verify the correctness of the model, it is necessary to make some experiments to simulate, and the simulation results are compared with the experimental results to verify the model. Wormhole expansion experiments are conducted in radial cores using real formation cores obtained from carbonate rock in Longwangmiao zone Sichuan of China, and the experiments’ results are shown in Figure 17. The data required in the simulation are taken from the experimental data of tardy et al. [33], as is shown in Figure 18, for example, the outer diameter and inner diameter of the core are 7.04 cm and 0.32 cm, respectively.

Different porosity distributions will produce different wormhole expansion patterns. Although random porosity distribution is mostly used to simulate in the literature, a large number of experimental results show that the porosity value of carbonate rocks conforms to the normal distribution law of spatial correlation. To study the influence of different porosity distribution laws on wormhole expansion, two porosity distributions are used in the simulation: random distribution porosity and spatially related porosity distribution. Figure 3 shows the wormhole morphology under the spatial correlation distribution and random distribution of porosity. It can be seen that although only one main wormhole breaks through the core, the dissolution forms of different porosity distributions are quite different. For the spatial correlation porosity distribution, there is only one relatively developed wormhole in the rock core, while other wormholes are relatively short and relatively undeveloped. For random porosity distribution, there are several relatively developed wormholes in the rock core at the same time. This is because the heterogeneity of spatially correlated porosity distribution is stronger than that of random porosity distribution. When the gelling acid is injected into the highly heterogeneous rock core, the gelling acid will produce uneven competition at the inlet and tend to enter the hole with the lowest resistance to produce wormholes. Once the wormhole is generated, most of the gelling acid will enter the wormhole, and then the main wormhole will be generated, resulting in the lack of development in other later-formed wormholes. For randomly distributed porosity, gelling acid liquid produces uniform competition at the inlet, resulting in multiple wormholes of the same size. Compared to the experimental results of Tardy et al., it is obvious that the wormhole morphology under the spatially correlated porosity distribution is similar to the experimental results, which verifies the correctness of the model.

## Data Availability

Data are contained within the article.
